# Shared learning in an interconnected world: innovations to advance global health equity

**DOI:** 10.1186/1744-8603-9-37

**Published:** 2013-08-30

**Authors:** Agnes Binagwaho, Cameron T Nutt, Vincent Mutabazi, Corine Karema, Sabin Nsanzimana, Michel Gasana, Peter C Drobac, Michael L Rich, Parfait Uwaliraye, Jean Pierre Nyemazi, Michael R Murphy, Claire M Wagner, Andrew Makaka, Hinda Ruton, Gita N Mody, Danielle R Zurovcik, Jonathan A Niconchuk, Cathy Mugeni, Fidele Ngabo, Jean de Dieu Ngirabega, Anita Asiimwe, Paul E Farmer

**Affiliations:** 1 Ministry of Health of Rwanda, Kigali, Rwanda; 2 Harvard Medical School, Boston, MA, USA; 3 Geisel School of Medicine at Dartmouth, Hanover, NH, USA; 4 Dartmouth Center for Health Care Delivery Science, Hanover, NH, USA; 5 Rwanda Biomedical Center, Kigali, Rwanda; 6 Partners In Health, Boston, MA, USA; 7 Brigham and Women’s Hospital, Boston, MA, USA; 8 MASS Design Group, Boston, MA, USA; 9 Global Health Delivery Partnership, Boston, MA, USA; 10 Massachusetts Institute of Technology, Boston, MA, USA

## Abstract

The notion of “reverse innovation”--that some insights from low-income countries might offer transferable lessons for wealthier contexts--is increasingly common in the global health and business strategy literature. Yet the perspectives of researchers and policymakers in settings where these innovations are developed have been largely absent from the discussion to date. In this Commentary, we present examples of programmatic, technological, and research-based innovations from Rwanda, and offer reflections on how the global health community might leverage innovative partnerships for shared learning and improved health outcomes in all countries.

## Introduction

In the early 2000s, citizens across Africa were mobilizing for access to the lifesaving antiretroviral therapy (ART) that had led to dramatic declines in AIDS mortality in the United States and Europe. Though the AIDS pandemic was crippling economies across the continent and countries faced the real prospect of losing an entire generation, many in public health placed effective treatment in opposition to prevention. Leading medical journals published modeling studies with confident assertions that flatly stated, for example, that “prevention is at least 28 times more cost-effective than ART [in Africa]” [1]; at least one influential voice in development circles argued that Africans living with HIV could never adhere to therapy because they “don’t know what Western time is [2].” These assertions, though un-buttressed by data, were typical of attitudes widespread among opinion makers in public health and within institutions charged with promoting health and economic development.

A decade later, in the wake of unprecedented international solidarity and funding, more than 7.1 million women, men, and children are receiving ART in Africa [3]. New studies have shown ART to reduce the likelihood of HIV transmission by up to 96% [4], and pooled analyses have demonstrated that African patients exhibit significantly higher adherence to treatment than their North American counterparts [5]. In Rwanda, AIDS-related deaths have declined by 83.1% since 2000—even more steeply than the comparable post-ART period after 1996 in Europe and North America [6]. An estimated 83.3% percent of HIV-positive adults on ART in Rwanda are virally suppressed [7], and community-based approaches to HIV care delivery refined in Rwanda and Haiti are now being implemented in the United States [8].

Pathologies from AIDS to cancer do not discriminate along lines of nationality, yet the notion that poor countries’ experiences addressing such conditions might offer lessons for settings rich and poor alike has only recently been recognized in the medical literature [9-12]. Most discussion to date has termed such exchanges “reverse innovation,” [8] though some have argued for a more explicitly bi-directional framework that recognizes best practices regardless of where they emerge [13-15].

### Towards a learning health system

While numerous gaps still exist (especially with regards to human resources), Rwanda has made significant progress in recent years towards an enabling regulatory and academic environment for evidence-based health innovation [16]. Most recently, the Rwandan government adopted a national Health Sector Research Policy to guide work from clinical trials to operational and social science research [17]. Research based on local needs has been an engine of improvement in the health sector, and has contributed to Rwanda’s likely achievement of the health-related Millennium Development goals [18,19].

We contend that Rwanda’s linkage of equitable health care delivery to research has catalyzed health innovation through three specific mechanisms. First, major health financing and care delivery initiatives have been evaluated through carefully planned phased-rollouts and investigational designs that students of innovation call “disciplined experiments” [20] (Table 1). Such approaches can be challenging to plan and controversial when they inevitably threaten conventional ways of doing things, but they are invaluable for learning and improving.

**Table 1:** “Disciplined experiments” to learn from, improve, and scale innovations in care delivery

•	Community-based health insurance scale-up [21,22]
•	Performance-based financing for maternal and child health services [23] and HIV care delivery [24]
•	Accompaniment for HIV patients by community health workers [25,26]
•	Comprehensive quality improvement and integrated platforms of community-based district health services [27]

A second and more routine approach to studying and disseminating innovations has been individual policymakers’ commitment to (and their institutions’ support for) retrospectively evaluating novel strategies to improve service delivery. Countries across Africa—and the world—face many of the same challenges, from treatment of multidrug-resistant tuberculosis patients co-infected with HIV to delivery of three doses of the human papillomavirus vaccine to adolescent girls. The innovations require rigorous monitoring and evaluation to objectively report their effect on the health system. Publication of Rwanda’s experiences in international peer-reviewed journals has shared solutions that worked in Rwanda with researchers across continents and contexts, and has sparked a nascent culture of innovation and reflection in the health sector (Table 2).

**Table 2:** Rigorous monitoring and evaluation of health systems innovations

•	“Diagonal approach” to HIV treatment rollout focused on health systems strengthening [28]
•	Task-shifting of HIV care and treatment from physicians to nurses [29] and community health workers [30]
•	Provision of food and transportation assistance to support adherence among multidrug-resistant tuberculosis patients [31]
•	School-based vaccination and community health worker tracing of out-of-school girls for Africa’s first human papillomavirus vaccination program [32]
•	Leveraging local expertise to increase enrollment in the health insurance system [33]
•	Mobile phone- and internet-based monitoring, evaluation, and reporting system for HIV diagnosis, care, and treatment [34,35]
•	The “Single Project Implementation Unit” to coordinate donor funds, and financial mechanisms for the provision of technical assistance to other low-income countries [36]
•	A “Mentoring and Enhanced Supervision of Health Centers” strategy to improve the quality of care delivered by nurse-providers at rural health centers [37]
•	Intervention coverage and quality improvement monitoring of national malaria [38] and integrated TB-HIV programs [39]
•	Collaborations with traditional healers to explore possible therapeutic efficacy of herbal medicines [16]

Finally, Rwanda has sought to provide a supportive environment for the development of new health tools designed specifically for the rural African context (Table 3). When distributed equitably, new technologies can accelerate health gains and narrow inequalities [15]. Public sector authorities encourage local manufacturing of devices developed or studied in Rwanda, and the World Bank recently ranked Rwanda 8^th^ of 185 countries for ease of starting a business and the 2^nd^ most improved business reformer since 2005 [40]. Although much work remains to fabricate the majority of medical innovations in-country, the breadth of available processes and increasing attention to international quality standards are promising.

**Table 3:** Supportive environment for context-specific health technology development

•	The “Byumba fix,” a low-cost, locally manufactured external fixator for setting fractures developed in the aftermath of the 1994 genocide [41]
•	Open MRS, a customizable open-source electronic medical record system [42]
•	PrePex, a nonsurgical male circumcision device for HIV prevention [43]
•	The Wound Pump, a low-cost negative pressure wound pump requiring no electricity [44]
•	mChip, a low-cost mobile phone-based HIV diagnostic device [45]

### Mutual capacity building

New technologies and better ways of organizing services will matter little to patients, however, if there is no one to deliver them. As a result of the 1994 genocide, Rwanda faces one of the world’s most severe human resources for health shortages. International partnerships, whether with non-governmental organizations or universities, are mandated to prioritize the transfer of capacity; over the past decade, however, it has become increasingly clear that the flow of knowledge goes—and, we argue, *must* go—the other way, too.

As one example, eight years of collaboration between the Rwandan Ministry of Health, the non-governmental organization Partners In Health, Harvard Medical School, and the Brigham and Women’s Hospital in Boston has contributed to the launch of a new discipline of global health delivery [46]. With courses now taught in both Boston and rural Rwanda, and with masters-level programs launching in both settings, stakeholders in both nations have built capacity among students, clinicians, and faculty while accelerating pedagogical innovation [47]. Rwanda’s Butaro District Hospital, built through a governmental partnership with MASS Design Group, Partners In Health, and the Clinton Health Access Initiative, is the site of design and infection control innovations that are being studied by architects as a model for contextually responsive, impact-oriented design, which could inform the way facilities are built to meet real needs globally, in both poor and wealthy nations [48,49].

On a much larger scale, Rwanda launched its Human Resources for Health Program in 2012, partnering with 16 American academic medical centers to increase the quantity and skill level of Rwanda’s physicians, nurses, midwives, and health managers, while diversifying the health workforce’s skill mix over seven years [50]. Each participating American faculty member is contracted for a twelve-month period and “twinned” with a Rwandan counterpart; the resulting individual and institutional partnerships will bear fruit well beyond the program’s end by creating cycles of innovation and learning in both directions. Most importantly, clinical practice and health systems are being enhanced in transformative ways in some of Rwanda’s most remote districts [51].

### The future of innovation in global health

As we have previously observed, for too long the world waited as “pathogens like HIV jet[ted] around the world” while “their remedies remain[ed] stuck in customs [52].” For many individual diseases, this situation is changing rapidly for the better; yet the global trade in ideas and innovations in health care delivery remains stunted. Leading editors of and contributors to the global scientific commons are well positioned to disrupt the status quo and foster new channels of South-North and South-South communication through the medical literature, where the perspectives of researchers and policymakers from low-income settings are currently few and far between.

We therefore eagerly welcome *Globalization and Health’s* new series on innovation in global health systems as an encouraging start [12]. But what might it take to catalyze a truly equal dialogue aimed at shared learning across borders and across historical gradients of inequality? Based on our experiences working together in Rwanda’s health sector, we suggest five key ingredients below:

1. *How will it benefit the poorest?—*This fundamental question should be at the heart of all global health partnerships, whether for research, policy, or service delivery, and it should fuel conversations between implementing partners, funding institutions, IRBs, and policymakers.

2. *Asking questions that matter to patients—*Global health research agendas should be derived in part from *patients’* notions of what is most at stake—this means listening [53]. The provision of transportation fare support for HIV patients, for instance, might seem outside the realm of a traditional research partnership. Yet given the average family income in rural Africa, a monthly round-trip bus ticket to a far-away clinic poses a similar proportional financial burden as would a business-class airplane ticket from Boston to Los Angeles for a middle-class American family.

3. *Experimentation across contexts—*Learning collaboratives, both between diverse partners in one setting [54] and between international collaborators [55], can be a powerful innovation tool when combined with “disciplined experimentation [8].” Timely publication of both positive and negative findings that result is a must, and requires journals and their editors to prize such experiences.

4. *Open access for open dialogue—*When accessing a single journal article can cost an African physician in the public sector two days’ salary (and what of the health journalist, or nurse?), even the best innovations will fail to make an impact. Open access journals are essential for harnessing the benefits of shared innovation.

5. *Reciprocity and respect—*International health research has long been semi-colonial and extractive [56]; global health equity also means intellectual partnership with the goal of equity. True partnership in international research projects means having local co-principal investigators and equal contributions to and representation on scientific articles. Further, not all “reverse innovations” stem from economic scarcity; scientists and program managers in poor countries are endowed with creativity and cultural resources just like their rich-country counterparts, and partners on both sides have lessons to share.

Looking forward, we believe that linking humility and bold vision to scientific rigor is the surest route to value and equity in global health. Some of the leading challenges facing health systems around the world in the twenty-first century may not be amenable to innovations derived from other contexts, but many are: all countries face the task of providing universal access to high quality, high value care. For the still-nascent field of global health to advance, we must embrace two-way learning; after all, we live in one world, not three, and the communities where this journal’s online readers live are as surely on the globe as are Kigali or Boston [57].
